# The Human Antibody Response to the Surface of *Mycobacterium tuberculosis*

**DOI:** 10.1371/journal.pone.0098938

**Published:** 2014-06-11

**Authors:** Casey C. Perley, Marc Frahm, Eva M. Click, Karen M. Dobos, Guido Ferrari, Jason E. Stout, Richard Frothingham

**Affiliations:** 1 Department of Molecular Genetics and Microbiology, Duke University Medical Center, Durham, North Carolina, United States of America; 2 Human Vaccine Institute, Duke University Medical Center, Durham, North Carolina, United States of America; 3 Division of Surgical Sciences, Department of Surgery, Duke University Medical Center, Durham, North Carolina, United States of America; 4 Department of Microbiology, Immunology and Pathology, Colorado State University, Fort Collins, Colorado, United States of America; 5 Division of Infectious Diseases, Department of Medicine, Duke University Medical Center, Durham, North Carolina, United States of America; McGill University, Canada

## Abstract

**Background:**

Vaccine-induced human antibodies to surface components of *Haemophilus influenzae* and *Streptococcus pneumonia* are correlated with protection. Monoclonal antibodies to surface components of *Mycobacterium tuberculosis* are also protective in animal models. We have characterized human antibodies that bind to the surface of live *M. tuberculosis*.

**Methods:**

Plasma from humans with latent tuberculosis (TB) infection (n = 23), active TB disease (n = 40), and uninfected controls (n = 9) were assayed by ELISA for reactivity to the live *M. tuberculosis* surface and to inactivated *M. tuberculosis* fractions (whole cell lysate, lipoarabinomannan, cell wall, and secreted proteins).

**Results:**

When compared to uninfected controls, patients with active TB disease had higher antibody titers to the surface of live *M. tuberculosis* (Δ = 0.72 log_10_), whole cell lysate (Δ = 0.82 log_10_), and secreted proteins (Δ = 0.62 log_10_), though there was substantial overlap between the two groups. Individuals with active disease had higher relative IgG avidity (Δ = 1.4 to 2.6) to all inactivated fractions. Surprisingly, the relative IgG avidity to the live *M. tuberculosis* surface was lower in the active disease group than in uninfected controls (Δ = –1.53, p = 0.004). Patients with active disease had higher IgG than IgM titers for all inactivated fractions (ratios, 2.8 to 10.1), but equal IgG and IgM titers to the live *M. tuberculosis* surface (ratio, 1.1). Higher antibody titers to the *M. tuberculosis* surface were observed in active disease patients who were BCG-vaccinated (Δ = 0.55 log_10_, p = 0.008), foreign-born (Δ = 0.61 log_10_, p = 0.004), or HIV-seronegative (Δ = 0.60 log_10_, p = 0.04). Higher relative IgG avidity scores to the *M. tuberculosis* surface were also observed in active disease patients who were BCG-vaccinated (Δ = 1.12, p<0.001) and foreign-born (Δ = 0.87, p = 0.01).

**Conclusions/Significance:**

Humans with active TB disease produce antibodies to the surface of *M. tuberculosis* with low avidity and with a low IgG/IgM ratio. Highly-avid IgG antibodies to the *M. tuberculosis* surface may be an appropriate target for future TB vaccines.

## Introduction

Tuberculosis (TB) is among the leading causes of death from infectious disease. Approximately one- third of the global population is infected with the causative agent, *Mycobacterium tuberculosis*. Around 8.8 million cases of active disease arose and 1.4 million deaths occurred in 2011 [1]. Resistance against the bacterium in mice and primates is primarily mediated by CD4+, and to a lesser extent CD8+ T-cells [2]–[4]. Humans with defects in interferon-γ signaling, a primary effector cytokine of T-cells, or those with depleted CD4+ T-cell numbers due to HIV infection, have increased susceptibility to mycobacterial infections [5], [6].

The role of antibodies in tuberculosis disease is widely debated. Early human studies focused on administration of sera, which was presumed to contain protective factors (antibodies), but the therapeutic effect of such administration was unclear [7]. More recent animal studies have demonstrated a protective role for both Fc receptors [8], [9] and IgA or IgG monoclonal antibodies administered prior to infection [10], [11]. Identifying the types of antibodies produced during *M. tuberculosis* infection, especially those against surface components, is important as these antibodies could potentially modify the course of infection.

Studies on antibody production against specific *M. tuberculosis* proteins abound, largely focused on their use in the diagnosis of either TB infection or TB disease. We have developed a novel whole-cell ELISA assay to detect antibodies to the live *M. tuberculosis* surface, and have compared these antibodies to those produced against a variety of inactivated antigenic fractions. Antibodies to the surface of live *M. tuberculosis* are present at low levels in uninfected individuals. Their titers increase in those with either latent TB infection or active TB disease. Paradoxically, IgG antibodies to the live *M. tuberculosis* surface have lower relative avidity in persons with active TB disease than in uninfected individuals. Antibodies to the *M. tuberculosis* surface are comprised of similar amounts of IgG and IgM, while antibodies to antigenic fractions are predominately IgG. Among patients with active disease, *M. tuberculosis* surface-binding antibodies have higher titers and higher relative IgG avidity scores in those who were BCG-vaccinated or foreign-born. These correlates were not seen for antibodies to the inactivated antigenic fractions.

Humoral immunity is increasingly recognized as a component of the protective immune response to *M. tuberculosis*, and as a target for TB vaccines [12]. Antibodies that bind to the surface of live *M. tuberculosis* are a potentially protective class, and they have different properties from antibodies produced against other mycobacterial targets. In particular, most patients with TB infection and disease fail to produce highly-avid IgG antibodies to the surface of the live bacterium. These results have important implications for TB vaccine development.

## Methods

### Bacterial Cultures and Lysates

*M. fortuitum* (ATCC 6841), *M. bovis* Bacille Calmette-Guérin Danish (ATCC 35733), *M. avium* subsp. *avium* Chester 1901 (ATCC 25291), *M. tuberculosis* H37Rv (ATCC 25681), and *M. intracellulare* (ATCC 13950*)* were grown in 7H9 media supplemented with 10% OADC, 0.5% glycerol, 0.05% tyloxapol, at 37°C, in a shaking incubator at 100–130 rpm, to an optical density (OD) between 1.0–1.5 as measured by a Cell Density Meter (Biowave, CO8000). For preparation of protein lysates, cultures were centrifuged at 600 g for 5 minutes and pellets were washed twice with PBS, pH 7.4, containing 0.05% tyloxapol. Pellets were lysed by vortexing with 0.1 micron glass beads in the presence of 50 mM tris hydrochloride, 0.5 mM EDTA, 60 mM sodium phosphate 0.67% SDS, and protease inhibitors (Roche 11836170001). Lysates were clarified by centrifugation at 600 g, then again at 16,000 g. Protein concentrations were determined using BCA protein assay (Pierce 23227).

### Additional ELISA Antigens

*M. tuberculosis* H37Rv fractions including culture filtrate (NR-14825), cell wall (NR-14828), whole cell lysate (NR-14822), and lipoarabinomannan (LAM) (NR-14848) were obtained from Colorado State University through BEI resources.

### ELISA

Antigen fractions and lysates were diluted in CBC buffer (7.5 mM sodium carbonate, 17.4 mM sodium bicarbonate, pH 9.0) and plated at a concentration of 200 ng/well in 96 well high-binding plates (Costar 3590). For ELISA assays to the live bacterial surface, growing cultures of mycobacteria at OD of 0.7–1.5 were pelleted at 200 g for 10 minutes. Live bacterial pellets were resuspended in CBC buffer to an OD of 1.5–2.5 and approximately 5×10^7^ bacteria were plated per well. After overnight incubation of plates at 4°C, wells were washed with PBS, pH 7.4 containing 0.05% Tween-20 (PBST), then blocked with 5% nonfat dry milk in CBC buffer. PBST-washed plates were incubated overnight at 4°C with immune and control plasma diluted in PBST containing 5% nonfat dry milk. A 1∶128 dilution of human plasma from a patient with active TB disease was used as a positive control. PBST with 5% nonfat dry milk was used as a negative control. PBST washed plates were then incubated for 1 hour with a 1∶3000 dilution of either alkaline phosphatase conjugated goat-anti-human Ig (whole molecule) (Sigma A15431), IgG (Fc-specific) (Sigma A9544), or IgM (Fc-specific) (Sigma A5937). After the addition of 1 µg/ml para-nitrophenyl phosphate (PNP) solution in CBC buffer containing 1.1 mM magnesium chloride (CBC-Mg), color development was recorded when positive control (POS) reached an OD of 2.0 as measured at 405 nm by spectrophotometer. Titers were defined as the reciprocal dilution which produced an OD of twice background, and were determined by exponential interpolation.

The ELISA assay for the live bacterial surface had some intrinsic variability due to the nature of its antigen. Bacteria clump together and can wash off during early wash steps. When compared to ELISA assays against the antigenic fractions, a longer incubation was required for color development in the whole cell ELISA. This led higher backgrounds than those observed for ELISA assays against antigen fractions. Whole cell ELISAs were rejected if the background was greater than 0.125 for Ig whole molecule and IgG (Fc-specific) assays, or greater than 0.160 for IgM (Fc-specific) assays. After optimization, the assay demonstrated acceptable reproducibility as shown in **Figure S1 in File S1**. The reproducibility of the whole cell ELISA (r^2^ = 0.80) was similar to the whole cell lysate ELISA (r^2^ = 0.86), but less than the LAM ELISA (r^2^ = 0.94).

### Avidity ELISA

Antigenic fractions, protein lysates and live mycobacteria were plated as described above. After 4°C incubation overnight, plates were washed with PBST and blocked with 3% bovine serum albumin (BSA) in PBS-T. Plates were sequentially washed in PBST, incubated with immune and control plasma diluted at a 1∶64 in 0.5% BSA in PBST for two hours at room temperature, re-washed in PBST and incubated with between 0.01 and 6 M sodium thiocyanate (Sigma) diluted in 0.5% BSA in PBST for 15 minutes at room temperature. PBST-washed plates were then incubated overnight at 4°C in 0.5% BSA in PBST with a 1∶3000 dilution of alkaline-phosphate conjugated goat-anti-human IgG (Fc specific) (Sigma9544). The same 1∶128 dilution of plasma from a patient with active TB disease was used as a positive control. PBST with 0.5% BSA was used as a negative control. After a final PBST wash plates were incubated in 1 µg/mL PNP in CBC-Mg solution. Color development was recorded when POS control reached OD 2.0 as measured at 405 nm by spectrophotometer. The avidity scores are defined as the concentration of sodium thiocyanate where the OD is equal to 50% of the no sodium thiocyanate control value.

### Statistical Analysis

For comparison between groups with normal distributions either a two-tailed student’s t-test or ANOVA was used. A Mann-Whitney test was used to compare two groups with non-normal distributions. For comparison of groups containing categorical variables, a Chi-squared or Fisher’s exact test was used as appropriate. Correlations between two continuous variables were probed through linear regression with r and p-values reported. All statistical calculations were conducted using PRISM (Graphpad Software).

### Human Subjects

Patients with latent TB infection and active TB disease were enrolled in Durham and Wake Counties, North Carolina, as part of the “TB Epitope study.” Latent TB infection was defined by a positive interferon-gamma release assay (Quantiferon Gold In-Tube, Cellestis Inc, Valencia, CA) with no radiographic or clinical findings suggestive of active TB. Active TB disease was defined by culture-proven TB disease or a diagnosis of clinical TB. This group included patients with a recent TB diagnosis, patients who were in the midst of therapy and patients who had completed therapy in the past. Uninfected patients were HIV-seronegative, tuberculin skin test-negative, healthy volunteers with no history of BCG vaccination. Informed written consent was obtained from participants, and the study was approved by the Duke University Medical Center Institutional Review Board.

### Cytokine Profiling

The cytokine profiling data used in this study was first reported in Frahm et al. 2011 [13]. In brief, whole blood was collected from subjects and assayed by Quantiferon Gold In-Tube (Cellestis Inc, Valencia, CA) testing. Each sample was incubated with either (a) a mixture of 3 TB specific antigens (ESAT-6, CFP-10, and TB7.7), (b) a mitogen positive control, or (c) a negative control tube containing no antigens. Following stimulation supernatant was collected and assayed for 25 cytokines and chemokines (IL-1β, IL-1RA, IL-2, IL-2R, IL-4, IL-5, IL-6, IL-7, IL-8, IL-10, IL-12 p40/70, IL-13, IL-15, IL-17, TNF-α, IFN-α, IFN-γ, GM-CSF, MIP-1α, MIP-1β, IP-10, MIG, Eotaxin, RANTES, MCP-1) through a Human Cytokine 25-plex assay (Biosource, Camarillo, CA).

## Results

### Demographic Information of Human Population

The 71 patients in our study included healthy uninfected volunteers (n = 9), patients with latent TB infection (n = 23), and patients with active TB disease (n = 40). Demographic and clinical data are shown in **Table 1**. As expected, there were differences among the groups in several clinical characteristics: Male gender was less common in uninfected and latent TB infection groups as compared to the active TB infection group (33% and 48% vs 73%; p = 0.05 and 0.06 respectively, Fisher’s Exact Test). BCG vaccination was less common in the uninfected group than in the latent TB infection or the active TB disease group (0% vs. 39% and 43%; p = 0.04 and 0.02 respectively). Similarly, birth in the US was more common in uninfected group than in the latent TB infection or the active TB disease group (78% vs. 44% and 50%; p = 0.05 and 0.06 respectively).

**Table 1:** Demographic and clinical data.

Variable	Uninfected (n = 9)		Latent TB infection (n = 23)		Active TB disease (n = 40)	
	N	Percent* ormean ± SD	N	Percent ormean ± SD	N	Percent ormean ± SD
**Age (yrs)**	9	38±14	23	42±17	40	43±15
**Gender**						
** Male**	3	33%	11	48%	29	73%
** Female**	6	67%	12	52%	11	28%
**Race/ethnicity**						
** White**	6	67%	8	35%	7	18%
** White Hispanic**	1	11%	4	17%	11	28%
** Black**	2	22%	9	39%	17	43%
** Black Hispanic**	0	0%	0	0%	1	3%
** Asian**	0	0%	2	9%	4	10%
**HIV seropositivity**						
** HIV+**	0	0%	2	8%	7	18%
** HIV–**	9	100%	19	83%	33	83%
**Unknown**			2	8%		
**Diabetes**						
** Yes**			2	9%	6	15%
** No**			21	91%	34	85%
**Tobacco usage**						
** Yes**			7	30%	11	28%
** No**			16	70%	29	73%
**Alcohol consumption**						
** None consumed**			14	61%	26	65%
** 1–3 drinks/day**			5	22%	6	15%
** >3 drinks/day or** **binge**			4	17%	8	20%
**BCG vaccination history**						
** Yes**	0	100%	9	39%	17	43%
** No**	9	0%	14	61%	22	55%
** Unknown**	0	0%	0	0%	1	3%
**US-born**						
** Yes**	7	78%	10	44%	20	50%
** No**	1	11%	13	57%	20	50%
** Unknown**	1	11%	0	0%	0	0%
**PPD induration (mm)**	n/a	n/a	19	24±19	37	20±16
**Disease state**	n/a	n/a	n/a	n/a		
** Pulmonary**					22	55%
** Extra-pulmonary**					14	35%
** Both**					4	10%

*Percentages may not add to 100% due to rounding.

### Human Antibody Titers to the Surface of Live *M. tuberculosis* and to Inactivated Antigenic Fractions

**Figure 1:**
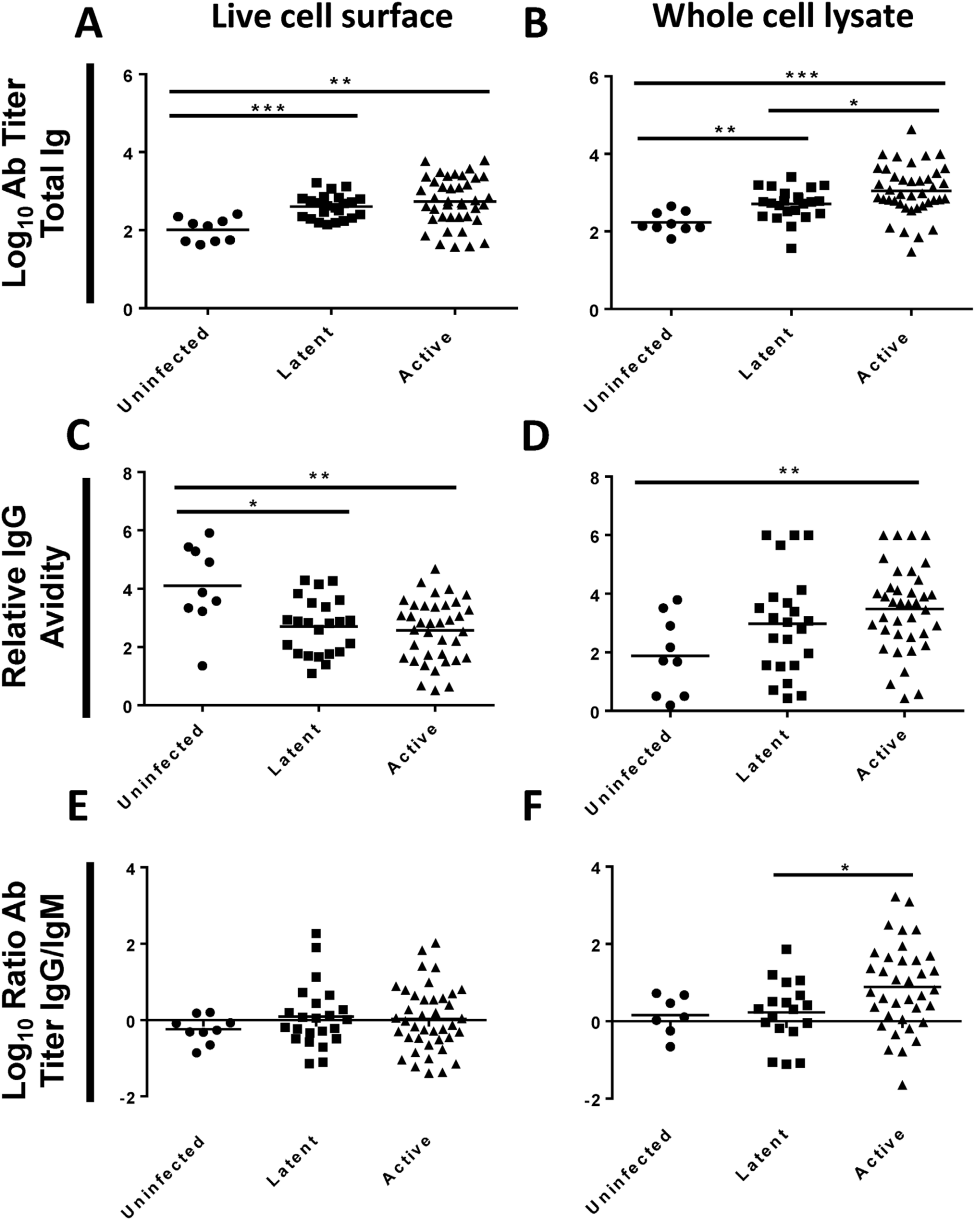
Antibody (Ab) responses to *M. tuberculosis* live cell surface or whole cell lysate. Plasma from PPD-negative volunteers (Uninfected), patients with latent TB infection (Latent), or patients with active TB disease (Active) were assayed by ELISA against the surface of live *M. tuberculosis* H37Rv or H37Rv whole cell lysate. (**A–B**) Log_10_ total Ig titers. (**C–D**) Relative IgG avidity. (**D–E**) Log_10_ ratio of IgG to IgM titers. Two-sided p-values by student’s t-test or Mann-Whitney test: (*) p≤0.05, (**) p≤0.01, (***) p≤0.001.

**Figure 2:**
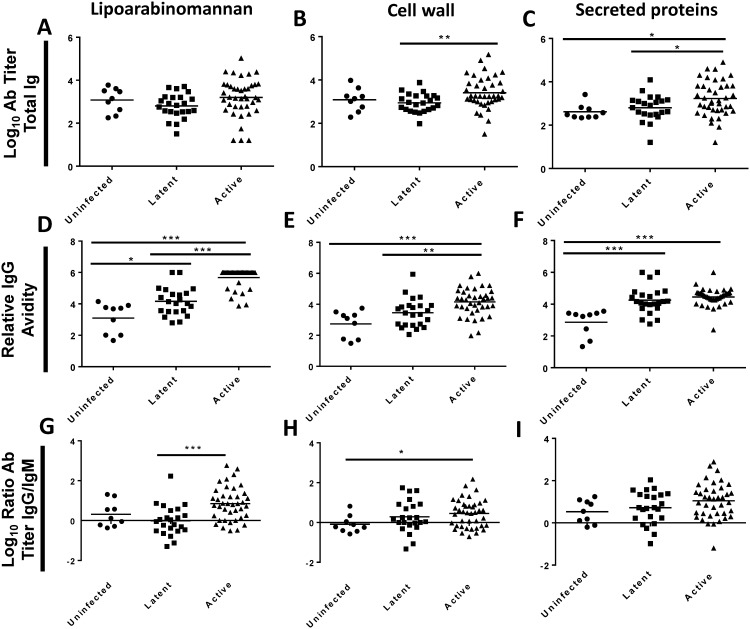
Antibody (Ab) responses to *M. tuberculosis* to lipoarabinomannan, cell wall, and secreted protein fractions. Plasma from PPD-negative volunteers (Uninfected), patients with latent TB infection (Latent), or patients with active TB disease (Active) were assayed by ELISA against the surface of live *M. tuberculosis* H37Rv or H37Rv whole cell lysate. (**A–C**) Log_10_ total Ig titers. (**D–F**) Relative IgG avidity. (**G–I**) Log_10_ ratio of IgG to IgM titers. Two-sided p-values by student’s t-test or Mann-Whitney test: (*) p≤0.05, (**) p≤0.01, (***) p≤0.001.

Plasma was assayed by ELISA to determine total antibody titers against the surface of live *M. tuberculosis* H37Rv and against four inactivated *M. tuberculosis* antigenic fractions (**Figure 1 A and B,** **Figure 2 A–C**). Plasma from the uninfected group reacted to the live *M. tuberculosis* surface (mean titer 2.0 log_10_) and to all four antigenic fractions (mean titers, 2.2 to 3.1 log_10_). Plasma from the active disease group had higher antibody titers to the surface of live *M. tuberculosis* (Δ = 0.72 log_10_), and to two of the antigenic fractions, whole cell lysate (Δ = 0.82 log_10_) and secreted proteins (Δ = 0.62 log_10_), when compared to uninfected controls. The latent TB infection group had intermediate titers, falling between the uninfected controls and the active TB disease groups for the surface of live *M. tuberculosis*, whole cell lysate and secreted proteins. Antibody titers to lipoarabinomannan and cell wall fractions were similar among the three patient groups.

Persons with active TB disease had variable responses to the live *M. tuberculosis* surface (**Figure 1 A**) with a range of 1.6 to 3.8 log_10_ and an interquartile range from 2.3 to 3.3 log_10_. Variability was generally less in the uninfected and latent infection groups. This variability resulted in overlap among the patient groups in total antibody titers in spite of statistically significant differences between groups (**Figure 1 A and B,** **Figure 2 A–C**). Antibody titers across antigens were correlated for each individual (data not shown).

### Relative IgG Avidity to the Surface of Live *M. tuberculosis* and to Inactivated Antigenic Fractions

ELISA was used to determine the relative IgG avidity to the live *M. tuberculosis* surface and to the four inactivated antigenic fractions. Repeated exposure to antigens, as in prime/boost vaccinations, normally leads to increased relative avidity (avidity maturation) [14]. As expected, persons with active disease had higher relative IgG avidity than the uninfected controls for all four inactivated fractions (Δ = 1.4 to 2.6, **Figure 1 D,** **Figure 2 D–F**), and these differences were statistically significant (p = 0.004 for whole cell lysate, and p<0.001 for the other fractions). Patients with latent TB infections had avidities intermediate between uninfected controls and active TB disease for all four fractions. Surprisingly, the relative IgG avidity to the live *M. tuberculosis* surface was actually lower in those with active TB disease (Δ = –1.53, p = 0.004), and in those with latent TB disease (Δ = –1.39, p = 0.011), when compared to uninfected controls (**Figure 1 C**).

### Ratio of IgG and IgM Antibodies to the Surface of Live *M. tuberculosis* and to Inactivated Antigenic Fractions

ELISA was used to measure the titers of IgG and IgM-specific antibodies to the live *M. tuberculosis* surface and to four inactivated antigenic fractions (**Figure 1 E and F,** **Figure 2 G–I**). Infectious agents typically generate an initial IgM response that is replaced by an IgG response [15]. Humans in all groups showed a mixed pattern of response with both IgG and IgM antibodies to the live *M. tuberculosis* surface, and to all four fractions. Patients in the uninfected and latent infection groups had roughly equal quantities of IgG and IgM to the *M. tuberculosis* surface, whole cell lysate, lipoarabinomannan, and cell wall (mean IgG/IgM ratios, −0.24 to 0.32 log_10_; **Figure 1 E and F,** **Figure 2 G–H**), and modest IgG predominance against secreted proteins (mean IgG/IgM ratios, 0.53 and 0.72 log_10_; **Figure 1 I**). Patients with active TB disease had higher IgG than IgM titers to all antigenic fractions (mean IgG/IgM ratios, 0.45 to 1.05 log_10_; **Figure 1 F,** **Figure 2 G–I**). In contrast, patients with active disease had equal amounts of IgG and IgM to the live *M. tuberculosis* surface (mean IgG/IgM ratio, 0.03 log_10_; **Figure 1 E**). In summary, patients with active TB disease mount atypical responses to the surface of live *M. tuberculosis* (low relative avidity, low IgG/IgM ratio), and this pattern is not seen for the inactivated fractions.

### Correlations between Antibody Titers and Clinical Characteristics

We explored potential correlations between antibody responses and clinical characteristics. **Table 2** displays correlations between clinical characteristics and total antibody titers to the live *M. tuberculosis* surface and to whole cell lysate for patients with active TB disease. For discrete clinical variables the log_10_ titer is reported (mean ± SEM). For continuous variables (age, PPD induration), the R-value is reported based on linear regression. P-values are not adjusted for multiple comparisons. Higher titers of *M. tuberculosis* surface-binding antibodies were observed in active TB disease patients who were BCG vaccinated versus unvaccinated (Δ = 0.55 log_10_, p = 0.008) or foreign born versus US born (Δ = 0.61 log_10_, p = 0.004). Race/ethnicity was also correlated with surface-binding antibody titers (p = 0.02), with white Hispanics and Asians possessing higher titers than other racial/ethnic groups. There was a high degree of overlap in these three variables, with many patients falling into one of two groups: BCG negative/US born/black or BCG positive/foreign born/non-black. HIV seropositivity was associated with lower surface-binding antibody titers (Δ = –0.60 log_10_, p = 0.039), as was increased age (r = –0.35, p = 0.027). Antibody titers to whole cell lysate showed weak trends in the same direction for several of these clinical characteristics (age, race/ethnicity, BCG vaccination, and foreign birth), but there were no statistically significant correlations (**Table 2**). Similar analyses are shown in **Table S1 in File S1** for antibody titers to lipoarabinomannan, cell wall, and secreted protein fractions. Increased age was associated with lower antibody titers to lipoarabinomannan (r = –0.35, p = 0.027) and no other statistically significant correlations were observed. Patients with latent TB infection showed no statistically significant correlations between antibody titers and clinical characteristics (**Table S2 in File S1, Table S3 in File S1**).

**Table 2:** Univariate correlation between clinical variables and total antibody titers to the live *M. tuberculosis* surface or to whole cell lysate in patients with active TB disease (n = 40).

Variable	Log_10_ total antibody titer by ELISA			
	*M. tuberculosis* surface		Whole cell lysate	
	R value ormean ± SEM	P value*	R value ormean ± SEM	P value
**Age**	R = –0.35	0.027	R = –0.21	0.19
**Gender**		0.89		0.67
** Male (n = 29)**	3.1±0.1		3.1±0.1	
** Female (n = 11)**	3.1±0.3		3.2±0.2	
**Race/ethnicity**		0.018		0.11
** White (n = 7)**	3.1±0.3		2.9±0.2	
** White Hispanic (n = 11)**	3.6±0.2		3.5±0.2	
** Black (n = 17)**	2.8±0.2		2.9±0.2	
** Black Hispanic (n = 1)**	2.7		2.0	
** Asian (n = 4)**	3.5±0.2		3.2±0.3	
**HIV seropositivity**		0.039		0.84
** HIV + (n = 7)**	2.6±0.2		3.1±0.2	
** HIV – (n = 33)**	3.2±0.1		3.0±0.1	
**Diabetes**		0.65		0.051
** Yes (n = 6)**	3.1±0.1		3.5±0.3	
** No (n = 34)**	3.2±0.3		3.0±0.1	
**Tobacco usage**		0.15		0.48
** Yes (n = 11)**	2.9±0.2		2.9±0.1	
** No (n = 29)**	3.3±0.1		3.1±0.1	
**Alcohol consumption**		0.87		0.76
** None consumed (n = 26)**	3.1±0.2		3.1±0.1	
** 1–3 drinks/day (n = 6)**	3.2±0.3		2.9±0.3	
** >3 drinks/day or binge (n = 9)**	3.2±0.2		3.1±0.1	
**BCG vaccination history**		0.008		0.087
** Yes (n = 17)**	3.4±0.1		3.2±0.1	
** No (n = 22)**	2.9±0.2		2.9±0.2	
**US-Born**		0.004		0.18
** Yes (n = 20)**	2.8±0.2		2.9±0.2	
** No (n = 20)**	3.4±0.1		3.2±0.2	
**PPD induration (mm)**	R = –0.18	0.28	R = –0.23	0.17
**Disease site**		0.96		0.20
** Pulmonary (n = 22)**	3.1±0.2		3.1±0.1	
** Extra-pulmonary (n = 14)**	3.1±0.2		2.8±0.2	
** Both (n = 4)**	3.1±0.4		3.4±0.2	

*P-value calculated using one-way ANOVA for categorical variables with >2 categories, students t-test for categorical variables with 2 categories and F-statistic for continuous variables. Welch’s correction was applied to the student’s t-test when variances were not equal between groups. All p-values are unadjusted for multiple comparisons.

### Correlations between Relative IgG Avidity and Clinical Characteristics

We conducted similar analyses for relative IgG avidity. **Table 3** displays correlations between clinical characteristics and relative IgG avidity to the live *M. tuberculosis* surface and to whole cell lysate for patients with active TB disease. The three clinical factors that correlated most strongly with surface-binding antibody titers, were also correlated with relative IgG avidity to the *M. tuberculosis* surface. Individuals with a history of BCG vaccination possessed higher relative IgG avidity to the surface of *M. tuberculosis*, than their non-vaccinated counterparts (Δ = 1.12, p<0.001), as did foreign born individuals (Δ = 0.87, p = 0.01). Race/ethnicity was also correlated with surface-binding antibody titers (p = 0.008), with white Hispanics and Asians possessing higher relative IgG avidity than other racial/ethnic groups. Taken together, the group of BCG-vaccinated/foreign born/non-black patients had higher antibody titers and higher relative IgG avidity to the surface of live *M. tuberculosis*. Relative IgG avidity to whole cell lysate was correlated with disease site (p = 0.018) with higher avidity in patients with extra-pulmonary disease. A similar trend was noted for higher relative IgG avidity to the live *M. tuberculosis* surface in patients with extra-pulmonary disease. No statistically significant correlations were observed for relative IgG avidity to lipoarabinomannan, cell wall, or secreted protein fractions (**Table S4 in File S1**). Higher relative IgG avidity to the surface of live *M. tuberculosis* was observed in patients with latent TB infection who consumed higher levels of alcohol (p = 0.045) and in those born in the US (Δ = 0.8, p = 0.033) (**Table S5 in File S1**). No other statistically significant correlations were observed between relative IgG avidity and clinical characteristics in patients with latent TB infection (**Table S5 in File S1, Table S6 in File S1**).

**Table 3:** Univariate correlation between clinical variables and relative IgG avidity of antibodies to the live *M. tuberculosis* surface or to whole cell lysate in patients with active TB disease (n = 40).

Variable	Relative IgG avidity			
	*M. tuberculosis* surface		Whole cell lysate	
	R value ormean ± SEM	P value*	R value ormean ± SEM	P value
**Age**	R = –0.13	0.46	R = –0.04	0.78
**Gender**		0.10		0.34
**Male (n = 29)**	2.7±0.2		3.6±0.3	
**Female (n = 11)**	2.1±0.4		3.1±0.3	
**Race/ethnicity**		0.008		0.71
**White (n = 7)**	1.8±0.4		4.0±0.4	
**White Hispanic (n = 11)**	3.0±0.3		3.2±0.5	
**Black (n = 17)**	2.3±0.2		3.3±0.4	
**Black Hispanic (n = 1)**	2.5		6.0	
**Asian (n = 4)**	3.9±0.4		3.4±0.4	
**HIV seropositivity**		0.30		0.15
**HIV + (n = 7)**	2.1±0.3		2.7±0.4	
**HIV – (n = 33)**	2.7±0.2		3.6±0.3	
**Diabetes**		0.30		0.27
**Yes (n = 6)**	2.1±0.4		2.8±1.0	
**No (n = 34)**	2.7±0.2		3.6±0.2	
**Tobacco usage**		0.64		0.31
**Yes (n = 11)**	2.7±0.4		3.9±0.4	
**No (n = 29)**	2.5±0.2		3.3±0.3	
**Alcohol consumption**		0.58		0.10
**None consumed (n = 26)**	2.5±0.2		3.1±0.2	
**1–3 drinks/day (n = 6)**	3.0±0.6		4.2±0.7	
**>3 drinks/day or binge (n = 9)**	2.5±0.2		4.1±0.7	
**BCG vaccination history**		<0.001		0.40
**Yes (n = 17)**	3.2±0.2		3.2±0.3	
**No (n = 22)**	2.1±0.2		3.8±0.4	
**US-Born**		0.010		0.80
**Yes (n = 20)**	2.1±0.2		3.5±0.4	
**No (n = 20)**	3.0±0.2		3.4±0.3	
**PPD induration (mm)**	R = –0.16	0.36	R = –0.10	0.57
**Disease site**		0.090		0.018
**Pulmonary (n = 22)**	2.5±0.3		3.0±0.3	
**Extra-pulmonary (n = 14)**	3.0±0.3		4.4±0.3	
**Both (n = 4)**	1.7±0.3		3.2±0.8	

*P-value calculated using one-way ANOVA for categorical variables with >2 categories, students t-test for categorical variables with 2 categories and F-statistic for continuous variables. Welch’s correction was applied to the student’s t-test when variances were not equal between groups. All p-values are unadjusted for multiple comparisons.

### Association of Cytokine Production with Surface-binding and Secreted Protein Antibody Titers and IgG Avidity Scores

All subjects with latent TB infection (n = 23) and a subset of subject with active TB disease (n = 10) were included in a previously published *M. tuberculosis* biomarker study [13]. Whole blood was incubated with *M. tuberculosis*-specific peptides and the concentrations of cytokines and chemokines determined. These values were plotted against surface-binding and whole cell lysate antibody titers (**Table S7 in File S1**) and IgG avidity scores (**Table S8 in File S1**), with r and p-values reported. In the ten patients with active TB disease, there was a positive correlation between IL-1β and antibody titers to the surface of live *M. tuberculosis* (r = 0.70, p = 0.02) and to whole cell lysate (r = 0.69, p = 0.03). The significance of this finding is unclear: since 96 pairwise comparisons were made, this rate of p-values below 0.05 would be expected by chance alone. When cytokines were grouped into those associated with either Th1 or Th2 responses, there was no pattern of association.

A greater number of cytokines were correlated with surface-binding antibody and whole cell lysate antibody IgG avidity in patients with active disease. There was a positive association between IFN-γ (r = 0.73, p = 0.02), IL-6 (r = 0.65, p = 0.04), IL-8 (r = 0.89, p = 0.001), MIP-1α (r = 0.77, p = 0.01) and MIP-1β (r = 0.85, p = 0.002) and surface-binding IgG avidity in patients with active disease. Associations were also observed between IL-10 (r = 0.77, p = 0.01), IL-13 (r = 0.65, p = 0.04), Eotaxin (r = –0.71, p = 0.02), and RANTES (r = –0.71, p = 0.02) and whole cell lysate IgG avidity in patients with active disease. No overall pattern of association was observed in association with either Th1 or Th2 cytokines.

### Antibody Titers and Avidity Scores to Environmental Mycobacteria

As shown in **Figure 1** **and** **Figure 2**, subjects with no evidence of TB infection had detectable antibody to the surface of live *M. tuberculosis* and to all four antigenic fractions derived from *M. tuberculosis.* Humans are exposed to environmental mycobacteria with significant antigenic similarity to *M. tuberculosis*. We generated protein lysates from *M. tuberculosis* H37Rv, and the type strains of *M. intracellulare, M. avium,* and *M. fortuitum*. We compared antibody titers to these lysates in our nine uninfected and a randomly selected group of ten patients with active TB disease (**Figure S2 A–D in File S1**). Uninfected patients had modest antibodies to lysates from all four mycobacterial species, and these titers were increased to all four species in all patients with active TB (Δ = 0.65 to 0.76 log_10_). Among those with active TB disease, antibody titers to the *M. tuberculosis* protein lysates were correlated to antibody titers to *M. avium* (r^2^ = 0.92, p<0.001), *M. intracellulare* (r^2^ = 0.94, p<0.001), and *M. fortuitum* (r^2^ = 0.58, p = 0.01) as shown in **Figure S2 E–G in File S1**.

**Figure 3:**
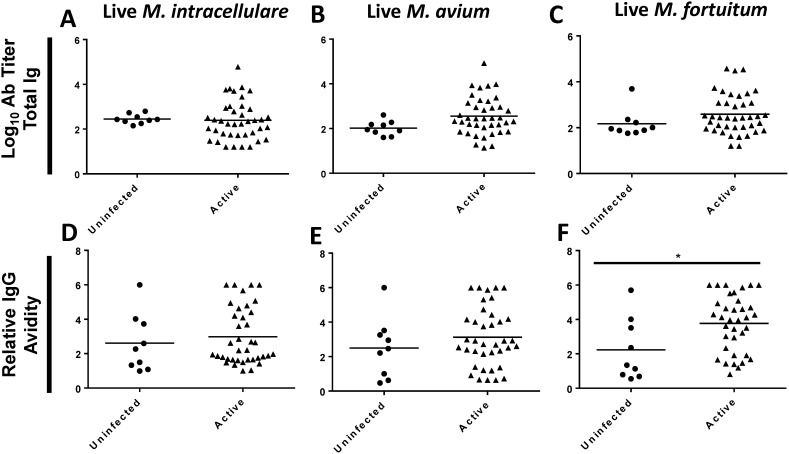
Reactivity of human plasma to surface of environmental mycobacteria. Plasma from PPD-negative volunteers (Uninfected), patients with latent TB infection (Latent), or patients with active TB disease (Active) were assayed by ELISA against the surface of live *M. intracellulare* (**A,D**) *M. avium* **(B,E**), and *M. fortuitum* **(C,F**). Log_10_ total Ig titers and relative IgG avidity are displayed. Two-sided p-values by student’s t-test or Mann-Whitney test: (*) p≤0.05, (**) p≤0.01, (***) p≤0.001.

As shown in **Figure 1 C**, relative IgG avidity to the surface of live *M. tuberculosis* was lower in persons with active TB disease than in uninfected patients. We measured antibody titers and relative IgG avidity to the surface of environmental mycobacteria to determine whether this reduced avidity is.

specific to *M. tuberculosis*. Antibody titers to the surface of *M. avium*, *M. intracellulare*, and *M. fortuitum* did not differ significantly between uninfected patients and patients with active TB disease (**Figure 3 A–C**). Relative IgG avidity did not differ between uninfected volunteers and individuals with active TB disease for the surface of live *M. intracellulare* (Δ = 0.37, p = 0.37) or the surface of live *M. avium* (Δ = 0.62, p = 0.33) (**Figure 3 D–E**). Patients with active TB disease had increased IgG avidity to the surface of live *M. fortuitum* (Δ = 1.54, p = 0.01) (**Figure 3 F**). These results contrast with the lower relative IgG avidity to the surface of *M. tuberculosis* seen in the same patients.

## Discussion

Previous work has characterized the human humoral response against *M. tuberculosis* by examining selected antigens to determine their utility for inclusion in diagnostic tests for tuberculosis, or as potential biomarkers [16]–[27]. Our results are in agreement with an overarching principle from these studies: Antibody titers in persons with latent TB infection or active TB disease are highly variable, and overlap with the titers found in uninfected individuals [25]–[28]. We observed a wide range of antibody titers among patients with active TB disease to the surface of live *M. tuberculosis* and to all four antigenic fractions (**Figure 1** **and** **Figure 2**). In each case, titers from patients with active TB disease overlapped with titers from uninfected subjects.

In addition our work has identified that antibodies to the surface of *M. tuberculosis* do not follow the same trends as antibodies against other *M. tuberculosis* antigens. While patients with active disease have slightly elevated titers compared to uninfected controls, there is a decrease in avidity, and no increase in the IgG/IgM ratio (**Figure 1** **)**, suggesting that there is a prolonged IgM response to the live surface of *M. tuberculosis*. This contrasts with antibodies to whole cell lysate, secreted proteins, and two components of the cell wall: LAM, and the cell wall fraction. The decrease of avidity to cell surface antigens may represent a lowering of overall avidity, conversion of antibodies from IgM to low-avidity IgG, or exhaustion of B-cells producing higher-avidity antibodies. The absence of increased avidity is notable none the less, as surface-binding antibodies are a potentially protective class, and humans lack high titer-high avidity antibodies against the surface. We identified three clinical factors (BCG vaccination, race/ethnicity, and country of origin) in persons with active TB disease that were strongly correlated with antibody titer (**Table 2**) and relative IgG avidity (**Table 3**) to the live *M. tuberculosis* surface. These factors were not correlated with antibody titers or relative IgG avidity to the inactivated antigenic fractions.

Numerous studies have examined the correlation of a variety of clinical factors with antibody production in tuberculosis and other diseases, though none have evaluated antibodies to the surface of live *M. tuberculosis*. While these studies have examined numerous factors found in this study including age [29], country of origin, BCG vaccination status and gender [30], HIV status [31], disease site [22], and PPD size [32], their results have varied depending on the antigen or cellular fraction used to quantitate antibody levels. Among persons with active TB disease, higher surface-binding antibody titers and higher relative IgG avidity were strongly correlated with BCG vaccination, foreign birth, and with race/ethnicity of either white Hispanic or Asian. Lower surface-binding antibody titers were associated with increasing age and with HIV seropositivity.

There was high overlap among the clinical variables of BCG vaccination, country of birth, and race/ethnicity among patient with active TB disease: 100% of BCG vaccinated patients were foreign born, 91% of BCG unvaccinated patients were US born, and 77% of patients identifying themselves as black were US born and BCG unvaccinated. This overlap was too high to permit meaningful multivariable analysis. The association with higher surface-binding antibodies may be primarily driven by one of these three factors, but the present data set does not define this factor. Other studies have examined race, BCG vaccination status, and country of origin in relation to antibody responses to antigenic fractions from *M. tuberculosis* [30], [33], [34]. Associations were noted with country of origin, but not with race or BCG vaccination. None of these studies evaluated antibodies to the surface of live *M. tuberculosis*.

Antibody titer and antibody avidity have been identified as correlates of protection for other bacterial vaccines including vaccines against *Haemophilus influenzae, Streptococcus pneumoniae*, and *Bordetella pertussis*. BCG vaccines may induce increased antibody titers and increased relative avidity to the surface of *M. tuberculosis*, and surface-binding antibodies may serve as a correlate of vaccine-induced protection. These questions could be tested using samples from randomized BCG vaccine trials.

Among active TB disease patients, lower surface binding antibody titers were associated with increasing age. A similar association was seen for antibodies to lipoarabinomannan, and trends were seen with other antigenic fractions. Elderly patients also have reduced antibody responses to vaccination [29]. Previous work focusing on TB has shown either a negative correlation between antibody titer and age, or no correlation at all depending on the specific antigens studied [22], [30].

Among patients with active TB disease, HIV seropositivity was associated with lower surface-binding antibody titers. Other studies have shown that HIV infection induces dysregulation in antibody responses to LAM, altering IgG subtype profiles, and decreasing the levels of IgG2 anti-*M. tuberculosis* antibodies as compared to HIV negative controls [31]. In both aging and HIV-infection, T-cells are also affected. Increased age is also known to lead to a collapse in T-cell diversity [35] and function [36], while HIV infection leads to a decrease in CD4+ T-cell count [37]. Older age and HIV infection are both associated with reactivation TB disease. While T-cells are primarily responsible for controlling *M. tuberculosis* infection [2]–[4], our results raise the possibility of a role for surface-binding antibodies as well.

We also examined cytokine levels in whole blood after TB-specific antigen stimulation for all patients with latent TB infection and ten active TB disease patients (**Table S7 in File S1** and **TableS8 in File S1**). A number of positive associations were found between TB-specific cytokine or chemokine production and IgG avidity to the *M. tuberculosis* surface, but no clear pattern of association was observed with either Th1 or Th2 cytokines. We had hypothesized that high antibody titer and avidity would correlate with elevated levels of Th2 cytokines, as these cytokines promote B-cell proliferation and differentiation to antibody-producing plasma cells [38]. Humans with active TB disease have also been demonstrated to generate Th2-like profiles in response to mycobacterial antigens [39]. However, studies in mice have demonstrated altered cytokine profiles over the course of infection, starting with a pro-inflammatory Th1 response, and switching to a Th0 balance (equal amounts of IL-2 and IL-4 production) during chronic infection [40].

We examined the antibody response to the surface of other live environmental mycobacteria to determine if the decreased avidity score observed to the surface of live *M. tuberculosis* was species-specific. As expected, antibodies from humans with active disease cross-reacted with protein lysates as well as the live surface of *M. avium*, *M. intracellulare*, and *M. fortuitum*. (**Figure 3** **, Figure S2 in File S1**). Previous studies have shown monoclonal antibodies produced against *M. tuberculosis* cross react with numerous other mycobacterial species including *M. kansasii, M. gastri, M. fortuitum* and *M. marinum* [41]. Notably, however, avidity scores did not decrease in individuals with active TB disease to the surface of live environmental microbes, indicating that the decreased avidity to the surface of *M. tuberculosis* is species-specific.

The cohort used for this study has several limitations which affect the scope of our conclusions. Due to the cross-sectional nature of this study, we cannot determine whether antibodies to the surface of *M. tuberculosis* reduce progression to disease in patients with latent TB infection, and we are unable to track changes in antibody levels associated with successful treatment. Prospective studies of patients with latent TB infection and active TB disease would provide additional insights. We did not collect data on parasitic infection. Some parasitic infections induce Th2-polarized adaptive immune responses, which could mask correlations between biomarkers and antibody titers/IgG avidity scores [42]. Our uninfected cohort was small in comparison to the other two groups, and it did not include BCG recipients. Despite the lower subject number, antibody titers and IgG avidity scores in the uninfected group had a similar range and standard deviation as the latent infection and active disease groups. With no BCG recipients in the uninfected group, we are unable to comment on the ability of the BCG vaccine to generate high titer/high avidity surface-binding antibodies in the absence of *M. tuberculosis* infection. Expansion of the uninfected cohort to include individuals with BCG vaccination, HIV seropositivity, and a great percentage of foreign born individuals would provide important information on the baseline surface-binding antibodies in a broader population.

Our ELISA assay also utilizes bacteria grown in liquid media in the presence of tyloxapol to reduce bacterial clumping. As tyloxapol has the potential to alter the bacterial surface [43], the surface of these culture-grown bacteria may differ from the surface of *M. tuberculosis* that is present in human tissues or sputum. Future studies detailing changes to the surface of the bacteria when grown *in vivo* versus *in vitro* will enable us to better characterize the human surface-binding antibody response to the most physiologically relevant form of the bacterium.

Natural TB infection in humans results in incomplete protection from re-infection [44], therefore, a fully effective vaccine may need to generate responses not found in infected humans. Antibodies to many microbes have the capacity to alter infection rate when they are present at the time of exposure [45]. Previous work has shown that murine antibodies, including some against surface components, are capable of altering the course of TB infection, if present at the time of *M. tuberculosis* challenge [11], [46]–[48]. Our work demonstrates that most humans with active TB infection lack high avidity surface-binding antibodies. Vaccines targeting specific antigenic components or epitopes that are accessible on the surface of *M. tuberculosis* may induce high-avidity surface-binding antibodies. Further research is warranted into the potential protective role of high-avidity antibodies to the *M. tuberculosis* surface.

In summary, these data demonstrate the difference between antibody responses to the surface of live *M. tuberculosis* and the responses to inactivated cellular fractions. When compared to uninfected humans, patients with active TB disease had increased avidity to cellular fractions, but decreased avidity to the *M. tuberculosis* surface. Patients with active TB disease had increased IgG/IgM ratios to cellular fractions but not to the *M. tuberculosis* surface. Higher surface-binding antibody titers were correlated with clinical factors associated with reduced rates of active TB disease: BCG vaccination, younger age, and HIV seronegativity. As highly-avid antibodies to surface components of other bacteria are associated with protection, the lack of high-avidity antibodies to the surface of *M. tuberculosis* in humans is notable.

## Supporting Information

File S1 **Figures S1 and S2, Tables S1–S8.** Figure S1. Reproducibility of human sera on different ELISA antigens: Human plasma was randomly selected from our cohort of 71 individuals and run in duplicate on (A) live *M. tuberculosis* cell surface (B) whole cell lysate and (C) lipoarabinomannan. Figure S2. Reactivity of human plasma to protein lysates from *M.* tuberculosis and environmental mycobacteria. Plasma from PPD-negative volunteers (Uninfected) and a randomly selected subset of patients with active TB disease (Active) were assayed against whole cell protein lysates from *M. tuberculosis* H37Rv (A), *M. intracellulare* (B), *M. avium* (C), and *M. fortuitum* (D). Two-sided p-values by student’s t-test: (*) p≤0.05, (**) p≤0.01, (***) p≤0.001. (E–G) Correlations between log_10_ antibody titers for *M tuberculosis* and environmental mycobacteria are displayed for individuals with active disease. Table S1. Univariate correlation between clinical variables and total antibody titers to lipoarabinomannan, cell wall, and secreted proteins in patients with active TB disease (n = 40). Table S2. Univariate correlation between clinical variables and total antibody titers to the live *M. tuberculosis* surface and to whole cell lysate in patients with latent TB infection (n = 23). Table S3. Univariate correlation between clinical variable and total antibody titers to lipoarabinomannan, cell wall, and secreted proteins for patients with latent TB infection (n = 23). Table S4. Univariate correlation between clinical variables and relative IgG avidity of antibodies to lipoarabinomannan, cell wall, and secreted proteins in patients with active TB disease (n = 40). Table S5. Univariate correlation between clinical variables and relative IgG avidity of antibodies to the live *M. tuberculosis* surface and to whole cell lysate in patients with latent TB infection (n = 23). Table S6. Univariate correlation between clinical factors and relative IgG avidity of antibodies to lipoarabinomannan, secreted proteins, and cell wall in patients with latent TB infection (n = 23). Table S7. Univariate correlation between cytokine levels in whole blood after Quantiferon-Gold peptide stimulation and total antibody titers to the live *M. tuberculosis* surface and to whole cell lysate in patients with latent TB infection or active TB disease. Table S8. Univariate correlation between cytokine levels in whole blood after Quantiferon-Gold peptide stimulation and relative IgG avidity to the live *M. tuberculosis* surface and to whole cell lysate in patients with latent TB infection or active TB disease.
(DOCX)
