# Supplementary material for: The Human Antibody Response to the Surface of Mycobacterium tuberculosis
Source: PLoS One. 2014 Jun 11;9(6):e98938. doi: 10.1371/journal.pone.0098938 (PMC4053328; doi:10.1371/journal.pone.0098938)
Supplement: File S1 — Figures S1 and S2, Tables S1–S8. Figure S1. Reproducibility of human sera on different ELISA antigens: Human plasma was randomly selected from our cohort of 71 individuals and run in duplicate on (A) live M. tuberculosis cell surface (B) whole cell lysate and (C) lipoarabinomannan. Figure S2. Reactivity of human plasma to protein lysates from M. tuberculosis and environmental mycobacteria. Plasma from PPD-negative volunteers (Uninfected) and a randomly selected subset of patients with active TB disease (Active) were assayed against whole cell protein lysates from M. tuberculosis H37Rv (A), M. intracellulare (B), M. avium (C), and M. fortuitum (D). Two-sided p-values by student’s t-test: (*) p≤0.05, (**) p≤0.01, (***) p≤0.001. (E–G) Correlations between log10 antibody titers for M tuberculosis and environmental mycobacteria are displayed for individuals with active disease. Table S1. Univariate correlation between clinical variables and total antibody titers to lipoarabinomannan, cell wall, and secreted proteins in patients with active TB disease (n = 40). Table S2. Univariate correlation between clinical variables and total antibody titers to the live M. tuberculosis surface and to whole cell lysate in patients with latent TB infection (n = 23). Table S3. Univariate correlation between clinical variable and total antibody titers to lipoarabinomannan, cell wall, and secreted proteins for patients with latent TB infection (n = 23). Table S4. Univariate correlation between clinical variables and relative IgG avidity of antibodies to lipoarabinomannan, cell wall, and secreted proteins in patients with active TB disease (n = 40). Table S5. Univariate correlation between clinical variables and relative IgG avidity of antibodies to the live M. tuberculosis surface and to whole cell lysate in patients with latent TB infection (n = 23). Table S6. Univariate correlation between clinical factors and relative IgG avidity of antibodies to lipoarabinomannan, secreted proteins, an [file pone.0098938.s001.docx]

**SUPPORTING INFORMATION**

**Perley CC. Frahm M, Click EM, Dobos KM, Ferrari G, Stout JE, Frothingham R. The human antibody response to the surface of *Mycobacterium tuberculosis***

**Whole cell lysate**

**Lipoarabinomannan**

**Live cell surface**

**Figure S1: Reproducibility of human sera on different ELISA antigens:** Human plasma was randomly selected from our cohort of 71 individuals and run in duplicate on (**A**) live *M. tuberculosis* cell surface (**B**) whole cell lysate and (**C**) lipoarabinomannan.

**Uninfected**

**Active**

***M. tuberculosis***

***M. intracellulare***

***M. avium***

***M. fortuitum***

**Uninfected**

**Active**

**Uninfected**

**Active**

**Uninfected**

**Active**

**Log_10_ Ab Titer**

**Total Ig**

**A**

**B**

**C**

**E**

**F**

**G**

**D**

**Figure S2: Reactivity of human plasma to protein lysates from *M.* tuberculosis and environmental mycobacteria.** Plasma from PPD-negative volunteers (Uninfected) and a randomly selected subset of patients with active TB disease (Active) were assayed against whole cell protein lysates from *M. tuberculosis* H37Rv (**A**), *M. intracellulare* (**B**), *M. avium* (**C**), and *M. fortuitum* (**D**). Two-sided p-values by student’s t-test: (*) p ≤ 0.05, (**) p ≤ 0.01, (***) p ≤ 0.001. (**E-G**) Correlations between log_10_ antibody titers for *M tuberculosis* and environmental mycobacteria are displayed for individuals with active disease.

| Variable | Log_10_ total antibody titer by ELISA | | | | | |
| --- | --- | --- | --- | --- | --- | --- |
|  | **Lipoarabinomannan** | | **Cell wall** | | **Secreted proteins** | |
|  | **R value or**  **mean ± SEM** | **P value*** | **R value or**  **mean ± SEM** | **P value** | **R value or**  **mean ± SEM** | **P value** |
| Age | R=-0.35 | 0.027 | R=-0.23 | 0.15 | R=-0.14 | 0.39 |
| Gender |  | 0.93 |  | 0.37 |  | 0.55 |
| Male (n=29) | 3.2 ± 0.2 |  | 3.3 ± 0.1 |  | 3.3 ± 0.2 |  |
| Female (n=11) | 3.2 ± 0.2 |  | 3.6 ± 0.3 |  | 3.1 ± 0.2 |  |
| Race/ethnicity |  | 0.059 |  | 0.48 |  | 0.31 |
| White (n=7) | 2.9 ± 0.4 |  | 3.4 ± 0.3 |  | 2.9 ± 0.2 |  |
| White Hispanic (n=11) | 3.8 ± 0.2 |  | 3.5 ± 0.1 |  | 3.6 ± 0.2 |  |
| Black (n=17) | 3.0 ± 0.2 |  | 3.2 ± 0.2 |  | 3.2 ± 0.3 |  |
| Black Hispanic (n=1) | 2.3 |  | 3.8 |  | 2.9 |  |
| Asian (n=4) | 3.2 ± 0.3 |  | 3.8 ± 0.4 |  | 3.3 ± 0.4 |  |
| HIV seropositivity |  | 0.10 |  | 0.60 |  | 0.16 |
| HIV + (n=7) | 2.7 ± 0.3 |  | 3.5 ± 0.3 |  | 2.9 ± 0.2 |  |
| HIV – (n=33) | 3.3 ± 0.3 |  | 3.4 ± 0.1 |  | 3.3 ± 0.1 |  |
| Diabetes |  | 0.35 |  | 0.70 |  | 0.12 |
| Yes (n=6) | 3.5 ± 0.5 |  | 3.5 ± 0.4 |  | 3.7 ± 0.3 |  |
| No (n=34) | 3.0 ± 0.4 |  | 3.4 ± 0.1 |  | 3.2 ± 0.1 |  |
| Tobacco usage |  | 0.61 |  | 0.67 |  | 0.33 |
| Yes(n=11) | 3.2 ± 0.2 |  | 3.5 ± 0.3 |  | 3.0 ± 0.2 |  |
| No (n=29) | 3.1 ± 0.2 |  | 3.4 ± 0.1 |  | 3.3 ± 0.1 |  |
| Alcohol consumption |  | 0.62 |  | 0.68 |  | 0.80 |
| None consumed (n=26) | 3.2 ± 0.2 |  | 3.4 ± 0.2 |  | 3.2 ± 0.1 |  |
| 1-3 drinks/day (n=6) | 3.0 ± 0.5 |  | 3.1 ± 0.2 |  | 3.2 ± 0.4 |  |
| >3 drinks/day or binge (n=9) | 3.4 ± 0.2 |  | 3.6 ± 0.2 |  | 3.4 ± 0.3 |  |
| BCG vaccination history |  | 0.057 |  | 0.18 |  | 0.24 |
| Yes (n=17) | 3.0 ± 0.2 |  | 3.6 ± 0.1 |  | 3.4 ± 0.2 |  |
| No (n=22) | 3.5 ± 0.2 |  | 3.2 ± 0.2 |  | 3.1 ± 0.2 |  |
| US-Born |  | 0.10 |  | 0.11 |  | 0.32 |
| Yes (n=20) | 3.4 ± 0.1 |  | 3.2 ± 0.2 |  | 3.1 ± 0.2 |  |
| No (n=20) | 3.0 ± 0.2 |  | 3.6 ± 0.1 |  | 3.4 ± 0.1 |  |
| PPD induration (mm) | R=-0.19 | 0.26 | R=-0.06 | 0.75 | R=-0.13 | 0.44 |
| Disease Site |  | 0.20 |  | 0.43 |  | 0.31 |
| Pulmonary (n=22) | 3.3 ± 0.2 |  | 3.4 ± 0.2 |  | 3.4 ± 0.2 |  |
| Extra-pulmonary (n=14) | 2.9 ± 0.2 |  | 3.2 ± 0.2 |  | 3.0 ± 0.2 |  |
| Both (n=4) | 3.6 ± 0.3 |  | 3.8 ± 0.5 |  | 3.5 ± 0.3 |  |

*P-value calculated using one-way ANOVA for categorical variables with >2 categories, students t-test for categorical variables with 2 categories and F-statistic for continuous variables Welch’s correction was applied to the student’s t-test when variances were not equal between groups. All p-values are unadjusted for multiple comparisons.

**Table S1**: **Univariate correlation between clinical variables and total antibody titers to lipoarabinomannan, cell wall, and secreted proteins in patients with active TB disease (n=40)**

**Table S2**: **Univariate correlation between clinical variables and total antibody titers to the live *M. tuberculosis* surface and to whole cell lysate in patients with latent TB infection (n=23)**

| **Variable** | **Log_10_ total antibody titer by ELISA** | | | | |
| --- | --- | --- | --- | --- | --- |
|  | ***M. tuberculosis* surface** | | **Whole cell lysate** | | |
|  | **R value or**  **mean ± SEM** | **P value*** | **R value or**  **mean ± SEM** | **P value‡** |  |
| **Age** | R=0.06 | 0.78 | R=0.25 | 0.24 |  |
| **Gender** |  | 0.66 |  | 0.12 |  |
| **Male (n=11)** | 3.0 ± 0.1 |  | 2.9 ± 0.1 |  |  |
| **Female (n=12)** | 2.9 ± 0.2 |  | 2.6 ± 0.1 |  |  |
| **Race/ethnicity** |  | 0.73 |  | 0.51 |  |
| **White (n=8)** | 2.9 ± 0.1 |  | 2.8 ± 0.1 |  |  |
| **White Hispanic (n=4)** | 3.1 ± 0.2 |  | 2.0 ± 0.4 |  |  |
| **Black (n=9)** | 3.0 ± 0.1 |  | 3.0 ± 0.1 |  |  |
| **Asian (n=2)** | 3.1 |  | 3.3 |  |  |
| **HIV seropositivity** |  | ND‡ |  | ND |  |
| **HIV + (n=2)** | 3.0 |  | 2.9 |  |  |
| **HIV – (n=19)** | 3.0 ± 0.1 |  | 3.0 ± 0.1 |  |  |
| **Diabetes** |  | ND |  | ND |  |
| **Yes (n=2)** | 3.1 |  | 3.0 |  |  |
| **No (n=21)** | 3.0 ± 0.1 |  | 2,7 ± 0.1 |  |  |
| **Tobacco usage** |  | 0.75 |  | 0.68 |  |
| **Yes(n=7)** | 3.0 ± 0.2 |  | 2.8 ± 0.1 |  |  |
| **No (n=16)** | 3.0 ± 0.1 |  | 2.7 ± 0.1 |  |  |
| **Alcohol consumption** |  | 0.29 |  | 0.39 |  |
| **None consumed (n=14)** | 2.9 ± 0.1 |  | 2.6 ± 0.1 |  |  |
| **1-3 drinks/day (n=5)** | 2.9 ± 0.1 |  | 2.7 ± 0.1 |  |  |
| **>3 drinks/day or binge (n=4)** | 3.2 ± 0.2 |  | 3.0 ± 0.1 |  |  |
| **BCG vaccination history** |  | 0.30 |  | 0.89 |  |
| **Yes (n=9)** | 3.1 ± 0.2 |  | 2.7 ± 0.2 |  |  |
| **No (n=14)** | 2.9 ± 0.1 |  | 2.7 ± 0.1 |  |  |
| **US-Born** |  | 0.86 |  | 0.76 |  |
| **Yes (n=13)** | 3.0 ± 0.1 |  | 2.7 ± 0.1 |  |  |
| **No (n=10)** | 2.9 ± 0.1 |  | 2.7 ± 0.2 |  |  |
| **PPD induration (mm)** | R=-0.18 | 0.46 | R=-0.11 | 0.64 |  |

*P-value calculated using one-way ANOVA for categorical variables with >2 categories, students t-test for categorical variables with 2 categories and F-statistic for continuous variables. Welch’s correction was applied to the student’s t-test when variances were not equal between groups. All p-values are unadjusted for multiple comparisons.

‡Not done. Pairwise comparisons were limited to clinical characteristics with at least three subjects in each group.

| Variable | Log_10_ total antibody titer by ELISA | | | | | | |
| --- | --- | --- | --- | --- | --- | --- | --- |
|  | **Lipoarabinomannan** | | **Cell wall** | | | **Secreted proteins** | |
|  | **R value or**  **mean ± SEM** | **P value*** | **R value or**  **mean ± SEM** | **P value** | | **R value or**  **mean ± SEM** | **P value** |
| Age | R=-0.22 | 0.32 | 0.18 | 0.42 | R=0.27 | | 0.23 |
| Gender |  | 0.89 |  | 0.32 |  | | 0.35 |
| Male (n=11) | 2.8 ± 0.2 |  | 3.3 ± 0.2 |  | 2.9 ± 0.2 | |  |
| Female (n=12) | 2.8 ± 0.2 |  | 3.7 ± 0.3 |  | 2.7 ± 0.2 | |  |
| Race/ethnicity |  | 0.21 |  | 0.80 |  | | 0.95 |
| White (n=8) | 2.5 ± 0.2 |  | 3.4 ± 0.2 |  | 2.7 ± 0.1 | |  |
| White Hispanic (n=4) | 3.1 ± 0.2 |  | 3.4 ± 0.5 |  | 3.0 ± 0.6 | |  |
| Black (n=9) | 2.9 ± 0.1 |  | 3.7 ± 0.4 |  | 2.8 ± 0.1 | |  |
| Asian (n=2) | 3.0 |  | 3.0 ± 0.4 |  | 2.9 | |  |
| HIV seropositivity |  | ND‡ |  | ND |  | | ND |
| HIV + (n=2) | 3.0 |  | 5.2 ± 0.7 |  | 2.5 | |  |
| HIV – (n=19) | 2.8 ± 0.1 |  | 3.3 ± 0.2 |  | 2.9 ± 0.1 | |  |
| Diabetes |  | ND |  | ND |  | | ND |
| Yes (n=2) | 3.1 |  | 3.0 ± 0.9 |  | 2.8 | |  |
| No (n=21) | 2.8 ± 0.2 |  | 3.5 ± 0.2 |  | 2.9 ± 0.1 | |  |
| Tobacco usage |  | 0.83 |  | 0.20 |  | | 0.99 |
| Yes(n=7) | 2.8 ± 0.2 |  | 3.8 ± 0.5 |  | 2.8 ± 0.2 | |  |
| No (n=16) | 2.8 ± 0.2 |  | 3.3 ± 0.2 |  | 2.8 ± 0.1 | |  |
| Alcohol consumption |  | 0.84 |  | 0.24 |  | | 0.98 |
| None consumed (n=14) | 2.8 ± 0.1 |  | 3.2 ± 0.2 |  | 2.9 ± 0.1 | |  |
| 1-3 drinks/day (n=5) | 2.7 ± 0.4 |  | 3.6 ± 0.2 |  | 2.8 ± 0.2 | |  |
| >3 drinks/day or binge (n=4) | 2.9 ± 0.1 |  | 4.1 ± 0.8 |  | 2.8 ± 0.2 | |  |
| BCG vaccination history |  | 0.68 |  | 0.70 |  | | 0.72 |
| Yes (n=9) | 2.9 ± 0.2 |  | 3.4 ± 0.3 |  | 2.9 ± 0.3 | |  |
| No (n=14) | 2.8 ± 0.2 |  | 3.5 ± 0.3 |  | 2.8 ± 0.1 | |  |
| US-Born |  | 0.84 |  | 0.26 |  | | 0.96 |
| Yes (n=13) | 2.8 ± 0.2 |  | 3.7 ± 0.3 |  | 2.8 ± 0.3 | |  |
| No (n=10) | 2.8 ± 0.2 |  | 3.2 ± 0.3 |  | 2.8 ± 0.1 | |  |
| PPD induration (mm) | R=0.06 | 0.80 | 0.07 | 0.78 | R=0.05 | | 0.83 |

*P-value calculated using one-way ANOVA for categorical variables with >2 categories, students t-test for categorical variables with 2 categories and F-statistic for continuous variables. Welch’s correction was applied to the student’s t-test when variances were not equal between groups. All p-values are unadjusted for multiple comparisons.

**Table S3: Univariate correlation between clinical variable and total antibody titers to lipoarabinomannan, cell wall, and secreted proteins for patients with latent TB infection (n=23)**

‡Not done. Pairwise comparisons were limited to clinical characteristics with at least three subjects in each group.

**Table S4**: **Univariate correlation between clinical variables and relative IgG avidity of antibodies to lipoarabinomannan, cell wall, and secreted proteins in patients with active TB disease (n=40)**

| Variable | Relative IgG Avidity | | | | | |
| --- | --- | --- | --- | --- | --- | --- |
|  | **Lipoarabinomannan** | | **Cell wall** | | **Secreted proteins** | |
|  | **R value or mean ± SEM** | **P value*** | **R value or mean ± SEM** | **P value** | **R value or mean ± SEM** | **P value** |
| Age | R=-0.01 | 0.59 | R=0.28 | 0.085 | R=0.04 | 0.80 |
| Gender |  | 0.36 |  | 0.75 |  | 0.40 |
| Male (n=29) | 5.6 ± 0.1 |  | 4.2 ± 0.2 |  | 4.5 ± 0.1 |  |
| Female (n=11) | 5.9 ± 0.2 |  | 4.1 ± 0.2 |  | 4.3 ± 0.3 |  |
| Race/ethnicity |  | 0.30 |  | 0.98 |  | 0.75 |
| White (n=7) | 6.0 |  | 4.2 ± 0.3 |  | 4.3 ± 0.1 |  |
| White Hispanic (n=11) | 5.4 ± 0.3 |  | 4.0 ± 0.2 |  | 4.4 ± 0.1 |  |
| Black (n=17) | 5.6 ± 0.2 |  | 4.2 ± 0.3 |  | 4.6 ± 0.2 |  |
| Black Hispanic (n=1) | 6.0 |  | 4.4 |  | 4.5 |  |
| Asian (n=4) | 6.0 |  | 4.1 ± 0.4 |  | 4.3 ± 0.3 |  |
| HIV seropositivity |  | 0.57 |  | 0.70 |  | 0.62 |
| HIV + (n=7) | 5.8 ± 0.2 |  | 4.0 ± 0.4 |  | 4.3 ± 0.1 |  |
| HIV – (n=33) | 5.6 ± 0.1 |  | 4.2 ± 0.2 |  | 4.5 ± 0.1 |  |
| Diabetes |  | 0.18 |  | 0.22 |  | 0.90 |
| Yes (n=6) | 5.8 ± 0.1 |  | 4.6 ± 0.4 |  | 4.5 ± 0.2 |  |
| No (n=34) | 5.4 ± 0.3 |  | 4.1 ± 0.2 |  | 4.4 ± 0.1 |  |
| Tobacco usage |  | 0.60 |  | 0.36 |  | 0.88 |
| Yes(n=11) | 5.6 ± 0.3 |  | 4.4 ± 0.4 |  | 4.4 ± 0.2 |  |
| No (n=29) | 5.7 ± 0.1 |  | 4.1 ± 0.1 |  | 4.5 ± 0.1 |  |
| Alcohol consumption |  | 0.17 |  | 0.72 |  | 0.58 |
| None consumed (n=26) | 5.7 ± 0.1 |  | 4.1 ± 0.2 |  | 4.4 ± 0.1 |  |
| 1-3 drinks/day (n=6) | 6.0 |  | 4.1 ± 0.5 |  | 4.4 ± 0.2 |  |
| >3 drinks/day or binge (n=9) | 5.3 ± 0.4 |  | 4.4 ± 0.4 |  | 4.5 ± 0.1 |  |
| BCG vaccination history |  | 0.82 |  | 0.93 |  | 0.52 |
| Yes (n=17) | 5.6 ± 0.2 |  | 4.2 ± 0.2 |  | 4.4 ± 0.1 |  |
| No (n=22) | 5.7 ± 0.1 |  | 4.2 ± 0.2 |  | 4.5 ± 0.1 |  |
| US-Born |  | 0.84 |  | 0.79 |  | 0.50 |
| Yes (n=20) | 5.7 ± 0.1 |  | 4.2 ± 0.2 |  | 4.5 ± 0.2 |  |
| No (n=20) | 5.7 ± 0.2 |  | 4.1 ± 0.2 |  | 4.4 ± 0.1 |  |
| PPD induration (mm) | R=-0.004 | 0.98 | R=0.18 | 0.30 | R=-0.18 | 0.30 |
| Disease Site |  | 0.55 |  | 0.21 |  | 0.75 |
| Pulmonary (n=22) | 5.7 ± 0.2 |  | 4.0 ± 0.2 |  | 4.4 ± 0.1 |  |
| Extra-pulmonary (n=14) | 5.6 ± 0.2 |  | 4.2 ± 0.2 |  | 4.4 ± 0.2 |  |
| Both (n=4) | 6.0 |  | 4.9 ± 0.2 |  | 4.7 ± 0.2 |  |

*P-value calculated using one-way ANOVA for categorical variables with >2 categories, students t-test for categorical variables with 2 categories and F-statistic for continuous variables Welch’s correction was applied to the student’s t-test when variances were not equal between groups.

| Variable | Relative IgG Avidity | | | | |
| --- | --- | --- | --- | --- | --- |
|  | ***M. tuberculosis* surface** | | **Whole cell lysate** | | |
|  | **R value or**  **mean ± SEM** | **P value*** | | **R value or**  **mean ± SEM** | **P value** |
| Age | R=0.33 | 0.12 | | R=0.10 | 0.64 |
| Gender |  | 0.49 | |  | 0.060 |
| Male (n=11) | 2.9 ± 0.2 |  | | 3.2 ± 0.5 |  |
| Female (n=12) | 2.6 ± 0.3 |  | | 2.8 ± 0.5 |  |
| Race/ethnicity |  | 0.53 | |  | 0.062 |
| White (n=8) | 2.8 ± 0.4 |  | | 2.5 ± 0.5 |  |
| White Hispanic (n=4) | 2.0 ± 0.5 |  | | 2.6 ± 0.7 |  |
| Black (n=9) | 2.9 ± 0.3 |  | | 3.0 ± 0.6 |  |
| Asian (n=2) | 3.0 ± 0.8 |  | | 6.0 |  |
| HIV seropositivity |  | ND‡ | |  | ND |
| HIV + (n=2) | 3.4 ± 0.8 |  | | 3.2 ± 2.2 |  |
| HIV – (n=19) | 2.6 ± 0.2 |  | | 3.0 ± 0.4 |  |
| Diabetes |  | ND | |  | ND |
| Yes (n=2) | 2.5 ± 0.8 |  | | 2.9 ± 0.1 |  |
| No (n=21) | 2.7 ± 0.2 |  | | 3.0 ± 0.4 |  |
| Tobacco usage |  | 0.11 | |  | 0.77 |
| Yes(n=7) | 3.2 ± 0.2 |  | | 2.8 ± 0.7 |  |
| No (n=16) | 2.5 ± 0.3 |  | | 3.0 ± 0.4 |  |
| Alcohol consumption |  | 0.045 | |  | 0.56 |
| None consumed (n=14) | 2.3 ± 0.2 |  | | 2.6 ± 0.5 |  |
| 1-3 drinks/day (n=5) | 3.4 ± 0.5 |  | | 3.5 ± 0.2 |  |
| >3 drinks/day or binge (n=4) | 3.2 ± 0.3 |  | | 3.4 ± 0.9 |  |
| BCG vaccination history |  | 0.45 | |  | 0.30 |
| Yes (n=9) | 2.5 ± 0.3 |  | | 2.5 ± 0.6 |  |
| No (n=14) | 2.8 ± 0.3 |  | | 3.3 ± 0.5 |  |
| US-Born |  | 0.033 | |  | 0.26 |
| Yes (n=13) | 3.0 ± 0.3 |  | | 3.4 ± 0.5 |  |
| No (n=10) | 2.2 ± 0.3 |  | | 2.5 ± 0.5 |  |
| PPD induration (mm) | R=0.004 | 0.99 | | R=0.14 | 0.56 |

*P-value calculated using one-way ANOVA for categorical variables with >2 categories, students t-test for categorical variables with 2 categories and F-statistic for continuous variables. Welch’s correction was applied to the student’s t-test when variances were not equal between groups. All p-values are unadjusted for multiple comparisons.

**Table S5**: **Univariate correlation between clinical variables and relative IgG avidity of antibodies to the live *M. tuberculosis* surface and to whole cell lysate in patients with latent TB infection (n=23)**

‡Not done. Pairwise comparisons were limited to clinical characteristics with at least three subjects in each group.

**Table S6: Univariate correlation between clinical factors and relative IgG avidity of antibodies to lipoarabinomannan, secreted proteins, and cell wall in patients with latent TB infection (n=23)**

| Variable | Relative IgG Avidity | | | | | | | |  |
| --- | --- | --- | --- | --- | --- | --- | --- | --- | --- |
|  | **Lipoarabinomannan** | | | **Cell wall** | | | **Secreted proteins** | |  |
|  | **R value or**  **mean ± SEM** | **P value*** | **R value or**  **mean ± SEM** | | **P value** | **R value or**  **mean ± SEM** | | **P value** | |
| Age | R=-0.33 | 0.13 | R=0.18 | | 0.42 | R=0.35 | | 0.10 | |
| Gender |  | 0.81 |  | | 0.32 |  | | 0.31 | |
| Male (n=11) | 4.2 ± 0.3 |  | 3.3 ± 0.2 | |  | 4.1 ± 0.2 | |  | |
| Female (n=12) | 4.1 ± 0.2 |  | 3.7 ± 0.3 | |  | 4.4 ± 0.3 | |  | |
| Race/ethnicity |  | 0.70 |  | | 0.80 |  | | 0.42 | |
| White (n=8) | 4.0 ± 0.4 |  | 3.4 ± 0.2 | |  | 4.3 ± 0.3 | |  | |
| White Hispanic (n=4) | 4.3 ± 0.6 |  | 3.4 ± 0.5 | |  | 3.8 ± 0.5 | |  | |
| Black (n=9) | 4.1 ± 0.3 |  | 3.7 ± 0.4 | |  | 4.5 ± 0.3 | |  | |
| Asian (n=2) | 4.8 ± 0.1 |  | 3.0 ± 0.4 | |  | 3.7 ± 0.3 | |  | |
| HIV seropositivity |  | ND‡ |  | | ND |  | | ND | |
| HIV + (n=2) | 4.8 ± 0.2 |  | 5.2 ± 0.7 | |  | 5.3 ± 1.1 | |  | |
| HIV – (n=19) | 4.0 ± 0.2 |  | 3.3 ± 0.2 | |  | 4.2 ± 0.2 | |  | |
| Diabetes |  | ND |  | | ND |  | | ND | |
| Yes (n=2) | 3.9 ± 1.1 |  | 3.0 ± 0.9 | |  | 4.0 ± 0.01 | |  | |
| No (n=21) | 4.2 ± 0.2 |  | 3.5 ± 0.2 | |  | 4.3 ± 0.2 | |  | |
| Tobacco usage |  | 0.51 |  | | 0.20 |  | | 0.54 | |
| Yes(n=7) | 4.4 ± 0.4 |  | 3.8 ± 0.5 | |  | 4.4 ± 0.2 | |  | |
| No (n=16) | 4.1 ± 0.2 |  | 3.3 ± 0.2 | |  | 4.2 ± 0.2 | |  | |
| Alcohol consumption |  | 0.61 |  | | 0.24 |  | | 0.11 | |
| None consumed (n=14) | 4.3 ± 0.2 |  | 3.2 ± 0.2 | |  | 4.0 ± 0.2 | |  | |
| 1-3 drinks/day (n=5) | 3.8 ± 0.5 |  | 3.6 ± 0.2 | |  | 4.7 ± 0.3 | |  | |
| >3 drinks/day or binge (n=4) | 4.0 ± 0.5 |  | 4.1 ± 0.8 | |  | 4.7 ± 0.4 | |  | |
| BCG vaccination history |  | 0.87 |  | | 0.70 |  | | 0.95 | |
| Yes (n=9) | 4.2 ± 0.3 |  | 3.4 ± 0.3 | |  | 4.2 ± 0.3 | |  | |
| No (n=14) | 4.1 ± 0.3 |  | 3.5 ± 0.3 | |  | 4.3 ± 0.2 | |  | |
| US-Born |  | 0.95 |  | | 0.26 |  | | 0.32 | |
| Yes (n=13) | 4.1 ± 0.3 |  | 3.7 ± 0.3 | |  | 4.4 ± 0.2 | |  | |
| No (n=10) | 4.1 ± 0.3 |  | 3.2 ± 0.3 | |  | 4.1 ± 0.3 | |  | |
| PPD induration (mm) | R=0.05 | 0.85 | R=0.07 | | 0.78 | R=0.15 | | 0.55 | |

*P-value calculated using one-way ANOVA for categorical variables with >2 categories, students t-test for categorical variables with 2 categories and F-statistic for continuous variables. Welch’s correction was applied to the student’s t-test when variances were not equal between groups. All p-values are unadjusted for multiple comparisons.

‡Not done. Pairwise comparisons were limited to clinical characteristics with at least three subjects in each group.

**Table S7: Univariate correlation between cytokine levels in whole blood after Quantiferon-Gold peptide stimulation and total antibody titers to the live *M. tuberculosis* surface and to whole cell lysate in patients with latent TB infection or active TB disease.**

|  | **Latent TB infection (n=23)** | | | | **Active TB Disease (n=10)** | | | |
| --- | --- | --- | --- | --- | --- | --- | --- | --- |
|  | ***M. tuberculosis s*urface Ab titer** | | **Whole cell lysate Ab titer** | | ***M. tuberculosis s*urface Ab Titer** | | **Whole cell lysate Ab titer** | |
|  | **R*** | **P value‡** | **R*** | **P value‡** | **R*** | **P value‡** | **R*** | **P value‡** |
| **Cytokines associated with a Th1 response** | | | | | | | | |
| IL-2 | -0.02 | 0.48 | -0.20 | 0.37 | 0.08 | 0.83 | -0.17 | 0.64 |
| IL-12p40/p70 | 0.004 | 0.78 | -0.22 | 0.32 | -0.06 | 0.86 | -0.55 | 0.10 |
| IFNγ | -0.01 | 0.68 | -0.13 | 0.54 | -0.12 | 0.74 | -0.55 | 0.10 |
| TNFα | -0.01 | 0.62 | -0.31 | 0.15 | 0.33 | 0.35 | 0.18 | 0.62 |
| **Cytokines associated with a Th2 response** | | | | | | | | |
| IL-4 | -0.02 | 0.50 | -0.10 | 0.65 | 0.26 | 0.47 | 0.12 | 0.75 |
| IL-5 | 0.01 | 0.73 | -0.20 | 0.36 | 0.35 | 0.32 | 0.09 | 0.80 |
| IL-6 | 0.000 | 0.92 | -0.34 | 0.11 | 0.52 | 0.12 | 0.40 | 0.26 |
| IL-10 | -0.04 | 0.37 | -0.11 | 0.61 | 0.28 | 0.43 | 0.01 | 0.99 |
| IL-13 | -0.002 | 0.85 | -0.03 | 0.87 | 0.00 | 0.99 | -0.32 | 0.37 |
| **Additional cytokines** | | | | | | | | |
| IL-1β | 0.002 | 0.84 | -0.34 | 0.11 | 0.70 | 0.02 | 0.69 | 0.03 |
| IL-1Rα | -0.02 | 0.56 | -0.16 | 0.45 | 0.31 | 0.39 | -0.06 | 0.87 |
| IL-2R | -0.005 | 0.76 | -0.20 | 0.37 | 0.03 | 0.93 | 0.09 | 0.80 |
| IL-7 | 0.000 | 0.95 | -0.08 | 0.74 | 0.10 | 0.79 | -0.15 | 0.67 |
| IL-8 | -0.16 | 0.06 | -0.30 | 0.17 | 0.45 | 0.20 | 0.00 | 1.00 |
| IL-15 | -0.01 | 0.72 | -0.01 | 0.96 | -0.37 | 0.30 | -0.44 | 0.21 |
| IL-17 | -0.004 | 0.77 | -0.14 | 0.52 | 0.42 | 0.22 | 0.00 | 1.00 |
| IFNα | -0.02 | 0.47 | -0.09 | 0.68 | 0.48 | 0.16 | 0.11 | 0.76 |
| GM-CSF | -0.001 | 0.87 | -0.26 | 0.23 | 0.58 | 0.08 | 0.15 | 0.69 |
| **Cytokines** | | | | | | | | |
| MIP-1α | -0.01 | 0.67 | -0.23 | 0.30 | 0.51 | 0.14 | 0.29 | 0.41 |
| MIP-1β | -0.06 | 0.26 | -0.16 | 0.47 | 0.47 | 0.18 | 0.07 | 0.85 |
| IP-10 | -0.03 | 0.42 | -0.14 | 0.51 | 0.08 | 0.82 | -0.22 | 0.54 |
| MIG | -0.03 | 0.44 | -0.04 | 0.87 | 0.24 | 0.51 | -0.27 | 0.44 |
| Eotaxin | 0.02 | 0.52 | -0.20 | 0.36 | 0.07 | 0.85 | 0.34 | 0.33 |
| RANTES | -0.13 | 0.09 | -0.31 | 0.15 | -0.47 | 0.17 | -0.25 | 0.49 |
| MCP-1 | 0.02 | 0.48 | -0.06 | 0.77 | 0.57 | 0.09 | 0.07 | 0.84 |

* R values determined by linear regression of the log-transformed surface or secreted antibody titers plotted against log-transformed cytokine concentrations (difference in cytokine concentrations between the TB and null antigen tubes).

‡ P-values determined by F-statistic. P-values are not adjusted for multiple comparisons.

**Table S8: Univariate correlation between cytokine levels in whole blood after Quantiferon-Gold peptide stimulation and relative IgG avidity to the live *M. tuberculosis* surface and to whole cell lysate in patients with latent TB infection or active TB disease.**

|  | **Latent TB infection (n=23)** | | | | **Active TB Disease (n=10)** | | | |  |  |  |  |  |  |  |  |
| --- | --- | --- | --- | --- | --- | --- | --- | --- | --- | --- | --- | --- | --- | --- | --- | --- |
|  | ***M. tuberculosis s*urface IgG avidity** | | **Whole cell lysate IgG avidity** | | ***M. tuberculosis s*urface IgG avidity** | | **Whole cell lysate IgG avidity** | |  |  |  |  |  |  |  |  |
|  | **R*** | **P value‡** | **R*** | **P value‡** | **R*** | **P value‡** | **R*** | **P value‡** |  |  |  |  |  |  |  |  |
| **Cytokines associated with a Th1 response** | | | | | | | | |  |  |  |  |  |  |  |  |
| IL-2 | 0.13 | 0.57 | 0.13 | 0.56 | 0.32 | 0.36 | 0.39 | 0.27 |  |  |  |  |  |  |  |  |
| IL-12p40/p70 | -0.25 | 0.24 | -0.07 | 0.75 | 0.45 | 0.19 | 0.10 | 0.79 |  |  |  |  |  |  |  |  |
| IFNγ | -0.29 | 0.17 | -0.24 | 0.28 | 0.73 | 0.02 | 0.38 | 0.28 |  |  |  |  |  |  |  |  |
| TNFα | 0.22 | 0.31 | -0.04 | 0.84 | 0.49 | 0.15 | 0.35 | 0.33 |  |  |  |  |  |  |  |  |
| **Cytokines associated with a Th2 response** | | | | | | | | |  |  |  |  |  |  |  |  |
| IL-4 | 0.06 | 0.77 | -0.04 | 0.87 | 0.31 | 0.38 | 0.01 | 0.98 |  |  |  |  |  |  |  |  |
| IL-5 | 0.12 | 0.58 | 0.29 | 0.17 | 0.22 | 0.55 | -0.09 | 0.80 |  |  |  |  |  |  |  |  |
| IL-6 | -0.33 | 0.12 | -0.23 | 0.29 | 0.65 | 0.04 | 0.03 | 0.94 |  |  |  |  |  |  |  |  |
| IL-10 | 0.00 | 1.00 | -0.12 | 0.59 | 0.40 | 0.26 | 0.77 | 0.01 |  |  |  |  |  |  |  |  |
| IL-13 | 0.19 | 0.40 | -0.09 | 0.67 | 0.56 | 0.09 | 0.65 | 0.04 |  |  |  |  |  |  |  |  |
| **Additional cytokines** | | | | | | | | |  |  |  |  |  |  |  |  |
| IL-1β | -0.34 | 0.12 | -0.12 | 0.58 | 0.32 | 0.37 | 0.50 | 0.14 |  |  |  |  |  |  |  |  |
| IL-1Rα | -0.19 | 0.40 | -0.02 | 0.95 | 0.39 | 0.27 | 0.13 | 0.72 |  |  |  |  |  |  |  |  |
| IL-2R | -0.32 | 0.14 | -0.20 | 0.36 | -0.35 | 0.32 | -0.07 | 0.85 |  |  |  |  |  |  |  |  |
| IL-7 | -0.02 | 0.93 | 0.32 | 0.17 | 0.50 | 0.14 | 0.13 | 0.73 |  |  |  |  |  |  |  |  |
| IL-8 | 0.15 | 0.50 | 0.06 | 0.78 | 0.89 | 0.001 | 0.13 | 0.73 |  |  |  |  |  |  |  |  |
| IL-15 | -0.14 | 0.51 | 0.20 | 0.37 | -0.07 | 0.85 | -0.49 | 0.15 |  |  |  |  |  |  |  |  |
| IL-17 | -0.02 | 0.93 | 0.39 | 0.07 | 0.32 | 0.37 | 0.06 | 0.86 |  |  |  |  |  |  |  |  |
| IFNα | 0.01 | 0.98 | -0.07 | 0.76 | 0.34 | 0.34 | -0.35 | 0.33 |  |  |  |  |  |  |  |  |
| GM-CSF | 0.40 | 0.06 | 0.61 | 0.00 | 0.54 | 0.10 | 0.06 | 0.87 |  |  |  |  |  |  |  |  |
| **Cytokines** | | | | | | | | |  |  |  |  |  |  |  |  |
| MIP-1α | -0.27 | 0.21 | -0.04 | 0.86 | 0.77 | 0.01 | 0.49 | 0.16 |  |  |  |  |  |  |  |  |
| MIP-1β | -0.10 | 0.65 | -0.01 | 0.96 | 0.85 | 0.002 | 0.44 | 0.20 |  |  |  |  |  |  |  |  |
| IP-10 | -0.06 | 0.78 | 0.09 | 0.68 | 0.27 | 0.45 | 0.09 | 0.81 |  |  |  |  |  |  |  |  |
| MIG | 0.19 | 0.38 | -0.05 | 0.82 | 0.64 | 0.05 | 0.53 | 0.12 |  |  |  |  |  |  |  |  |
| Eotaxin | -0.04 | 0.86 | 0.27 | 0.21 | -0.55 | 0.10 | -0.71 | 0.02 |  |  |  |  |  |  |  |  |
| RANTES | 0.05 | 0.83 | 0.06 | 0.79 | -0.27 | 0.44 | -0.71 | 0.02 |  |  |  |  |  |  |  |  |
| MCP-1 | 0.08 | 0.73 | -0.02 | 0.93 | 0.67 | 0.03 | 0.04 | 0.91 |  |  |  |  |  |  |  |  |

* R values determined by linear regression of the log-transformed surface or secreted antibody titers plotted against log-transformed cytokine concentrations (difference in cytokine concentrations between the TB and null antigen tubes).

‡ P-values determined by F-statistic. P-values are not adjusted for multiple comparisons.
